# Integrated network-diversity analyses suggest suppressive effect of Hodgkin’s lymphoma and slightly relieving effect of chemotherapy on human milk microbiome

**DOI:** 10.1038/srep28048

**Published:** 2016-07-08

**Authors:** Zhanshan (Sam) Ma, Lianwei Li, Wendy Li, Jie Li, Hongju Chen

**Affiliations:** 1 Computational Biology and Medical Ecology Lab, State Key Lab of Genetic Resources and Evolution, Kunming Institute of Zoology, Chinese Academy of Sciences, Kunming 650023, China; 2 College of Mathematics, Honghe University, Mengzi, Yunnan 661100, China

## Abstract

We aim to investigate the effects of Hodgkin’s lymphoma and the chemotherapy for treating the disease on the human milk microbiome through integrated network and community diversity analyses. Our analyses suggest that Hodgkin’s lymphoma seems to have a suppressing effect on the milk microbiome by lowering the milk microbial community diversity, as measured by the Hill numbers profiles. Although the *diversity* analysis did not reveal an effect of chemotherapy on community diversity, bacterial species interaction network analysis shows that chemotherapy may help to slightly restore the milk microbiome impacted by Hodgkin’s lymphoma through its influence on the interactions among species (or OTUs). We further constructed *diversity-metabolites* network, which suggests that the milk microbial diversity is positively correlated with some beneficial milk metabolites such as DHA (DocosaHexaenoic Acid), and that the diversity is negatively correlated with some potentially harmful metabolites such as Butanal. We hence postulate that higher milk microbial diversity should be a signature of healthy mothers and beneficial to infants. Finally, we constructed metabolites OTU correlation networks, from which we identified some special OTUs. These OTUs deserve further investigations given their apparent involvements in regulating the levels of critical milk metabolites such as DHA, Inositol and Butanal.

With the rapid expansions of metagenomics technology and human microbiome project (HMP)^1^,^2^, the milk microbiome has also received increasing attention in recent years^3^,^4^,^5^,^6^,^7^,^8^,^9^,^10^,^11^,^12^. These existing studies have demonstrated fundamental importance of milk microbiome in maintaining the nutritional and health values of breast milk to infants and mothers. For example, in a recent report, Urbaniak *et al*.^10^ called for the attention to the impact of drugs administrated to the mothers on the milk microbiome as well as the potential health consequences for the infants given the critical significance of milk contents in shaping infant development and immunity. They presented the first longitudinal study on the effects of chemotherapy on the milk microbiome and discovered that chemotherapy for Hodgkin’s lymphoma caused a significant deviation from a healthy microbial and metabolic profile, and led to the decline of beneficial metabolites including DHA (docosahexaenoic acid) and Inositol^10^. In this study, we further analyzed Urbaniak *et al*.^10^ microbial and metabolic datasets to answer some new questions, beyond the findings revealed in their original report, by applying ecological *network analysis* and *diversity profile* analysis. Specifically, we hope our analyses will shed important lights on the following four biomedical questions: (*i*) Do Hodgkin’s lymphoma and/or the chemotherapy for treating the disease add any significant ‘signature’ to the impacted milk microbiome compared with the healthy milk microbiome? (*ii*) Does the chemotherapy have significant influence on the milk microbial diversity? If no, does it have any other important impacts that were missed by the diversity analysis? (*iii*) How are the metabolites in the breast milk related to the milk microbial diversity? (*iv*) Are there any specific bacteria species (OTUs) that are more closely associated with the important metabolites than their peers? We also discuss the biomedical implications of these questions. At present, answering to these questions is of pressing urgency and, even if preliminary, is of great biomedical importance, given that the critical significance of milk components to the development and immunity of infants is still poorly understood, as reiterated in Urbaniak *et al*.^10^.

Methodologically, we realized that the traditional ecological analysis such as simple diversity index or multivariate analysis approaches such as PCA (principal component analysis) and PCoA (principal coordinate analysis) may be insufficient in answering the new questions raised above. New and more powerful approaches may be necessary to detect sophisticated patterns and answer those questions^23^. Specifically, we replace the traditional diversity measures such as species richness and Shannon index with more comprehensive *diversity profile*—the Hill numbers^13^,^14^,^15^,^16^,^17^,^18^,^19^. The *diversity profile* measured in a series of Hill numbers at different orders (*q* = 0, 1, 2, 3) is now well recognized as the most appropriate metric for measuring alpha diversity because it overcomes the deficiencies of the traditional single diversity index by unifying the units of diversity measures with the concept of “*number of species equivalents*” (*i.e*., the Hill numbers)^19^. Furthermore, by using a series of entropies at different nonlinearity orders (diversity order *q*), the information contained in the species abundance distribution (SAD) of a community is captured in the diversity profile, making the long debated issue on which diversity index is superior in the community ecology mostly obsolete. It has also been demonstrated that Hill numbers have highly desirable advantages in measuring beta diversity as well as community similarity thanks to its satisfaction with *replication* principle, especially with the multiplicative partition of beta diversity^16^. Another approach we use in this study is the network analysis, which has become a powerful tool in the arsenal of computational biologists during the last decade^20^,^21^,^22^,^23^, but has relatively not been widely applied to the studies of microbiome^23^. As it becomes clear in the subsequent sections that either diversity analysis or network analysis alone is not sufficient to address the biomedical questions we raised above, and an integrated analysis with both approaches is adopted in this report.

## Materials and Methods

### The breast milk microbiome and metabolome datasets

The 16S rRNA and metabolites datasets of the breast milk microbiomes, analyzed in this report, were first reported in Urbaniak *et al*.^10^, and a brief description is presented as follows. A series of longitudinal milk samples were collected from a lactating women undergoing chemotherapy for treating Hodgkin’s lymphoma every two weeks over a four-month period, and a cohort of 8 healthy women were sampled one time for their mature milk as control. With the longitudinal study, a total of 16 milk samples including 8 before chemotherapy and 8 after chemotherapy were collected. The datasets of 16S rRNA reads and corresponding OTU tables (97% cutoff of similarity) for the *pre-chemotherapy*, *post-chemotherapy* and *healthy* cohort samples were obtained by using the Ion Torrent platform and subsequent bioinformatics analysis. The metabolome datasets for the milk samples were obtained with gas chromatography-mass spectrometry. Detailed information on both datasets is referred to Urbaniak *et al*.^10^.

### The computational procedures for network analyses

Three types of correlation networks were constructed with standard network analysis techniques explained in Junker & Schreiber^22^, and these are: (*i*) The milk bacterial species interaction networks (SIN) were built based on the pair-wise correlation between OTU abundances, including three networks for pre-chemotherapy, post-chemotherapy, and healthy microbiome samples, respectively. To reduce the effect of potentially spurious OTU reads, we removed OTUs whose total reads in a treatment are less than 5 in the construction of SINs. (*ii*) The diversity-metabolites network (DMN) was built based on the pair-wise correlation between community diversities and metabolite abundances. (*iii*) The Metabolite-OTU interaction network (MON) was built based on the correlation between the metabolite abundance and OTU abundance. The correlation relationship was established based on Pearson’s correlation coefficients with *p*-value ≤ 0.05, and the actual computation was performed with Cytoscape software^20^ and iGraph package^24^. In addition, the MCODE plug-in^21^ for Cytoscape was utilized to detect clusters in the bacterial SINs.

### Community diversities in the Hill numbers

The Hill numbers, originally introduced as an *evenness* index in economics by Hill^13^ who was apparently inspired by Renyi’s^25^ general entropy of order *q*, has not received the attention it deserves in ecology until recent years. Chao *et al*.^15^,^16^ further clarified Hill’s numbers for measuring alpha diversity of biodiversity as:


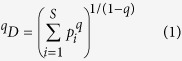


where *S* is the number of species or OTUs, *p*_i_ is the relative abundance of species *i*, *q* is the order number of diversity (*q* = 0, 1, 2, 3). The Hill numbers are in units of *species equivalents*, and measure the effective number of *species* or *species equivalents*.

The Hill number is undefined for *q* = 1, but its limit as *q* approaches to *1* exists in the following form:


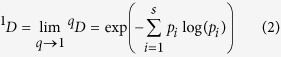


The parameter *q* determines the sensitivity of the Hill number to the relative frequencies of species abundances. When *q* = 0, the species abundances do not count at all and ^*0*^*D* = *S, i.e.,* species richness. When *q* = 1, ^*1*^*D* equal the *exponential* of Shannon entropy, and is interpreted as the number of typical or common species in the community. When *q* = 2, ^*2*^*D* equal the reciprocal of Simpson index, *i.e*.,


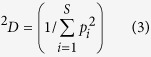


which is interpreted as the number of dominant or very abundant species in the community (Chao *et al*.^15^,^16^). The general interpretation of diversity of order *q* is that the community contains ^*q*^*D* = *x* equally abundant species. In this study, we compute the Hill numbers at order *q* = 0–4, for each of the *pre-chemotherapy*, *post-chemotherapy* and *healthy* cohort samples.

## Results and Discussion

### The bacterial species interaction network (SIN) in the pre-chemotherapy, post-chemotherapy and healthy milk microbiome samples

**Figure 1:**
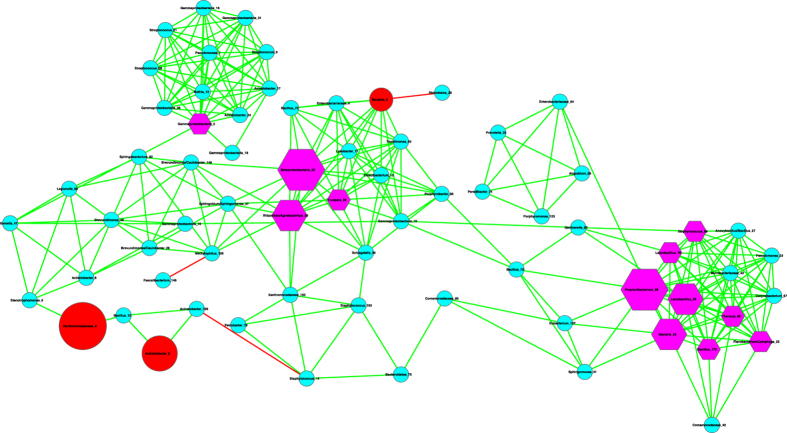
The milk bacterial species interaction network constructed with the pre-chemotherapy samples. Symbols used: edges in green—positive correlation, edges in red—negative correlation, hexagon in pink—hubs (nodes with the top three highest degrees), cycles in red—MAO (top three most abundant OTUs), and cycles in pink— assuming dual role of hub and MAO.

**Figure 2:**
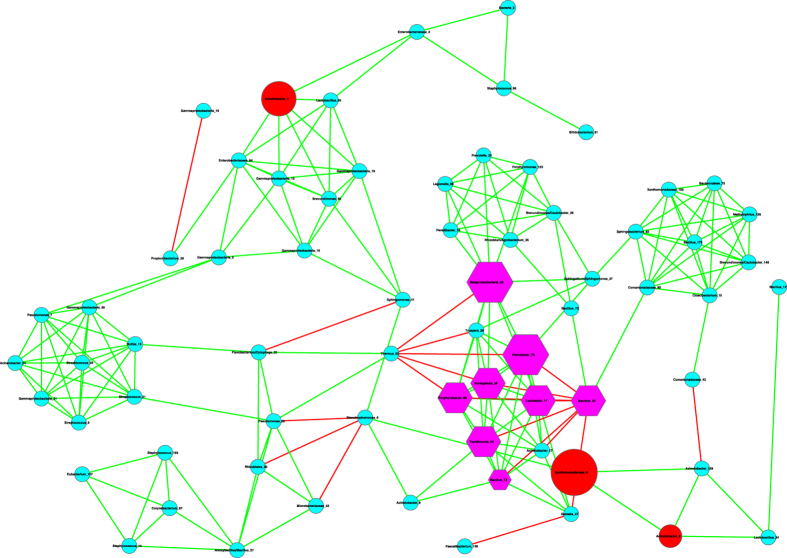
The milk bacterial species interaction network constructed with the post-chemotherapy samples. Symbols used: edges in green—positive correlation, edges in red—negative correlation, hexagon in pink—hubs (nodes with the top three highest degrees), cycles in red—MAO (top three most abundant OTUs), and cycles in pink— assuming dual role of hub and MAO.

**Figure 3:**
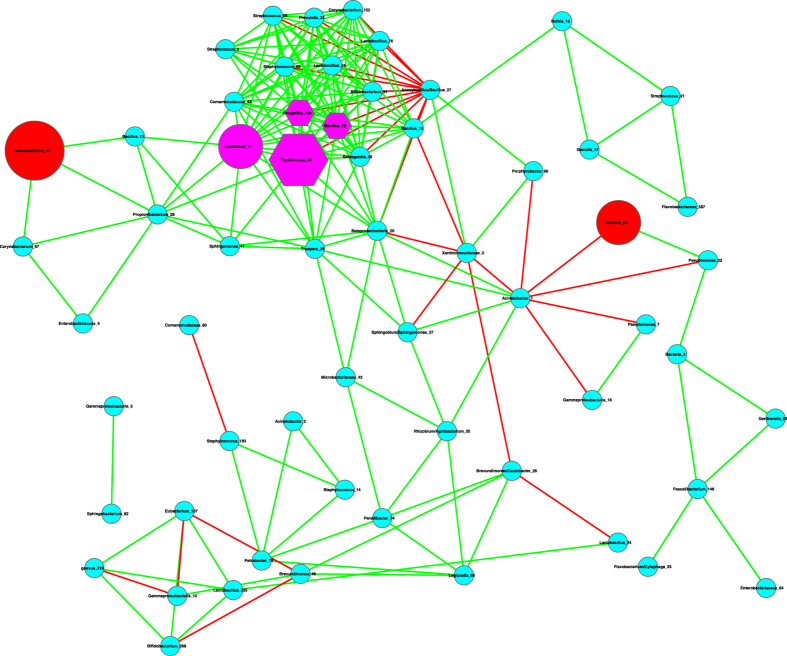
The milk bacterial species interaction network constructed with the healthy samples. Symbols used: edges in green—positive correlation, edges in red—negative correlation, hexagon in pink—hubs (nodes with the top three highest degrees), cycles in red—MAO (top three most abundant OTUs), and cycles in pink— assuming dual role of hub and MAO.

The basic network analysis performed with Cytoscape and iGraph software packages suggest the following findings (Tables 1, 2, Figs 1, 2, 3):

(*i*) Most network properties exhibited in Table 1 are similar between the healthy and diseased networks (the latter includes both *pre-* and *post-*chemotherapy networks) or between any of the three pairs. Nonetheless, one item in Table 1 (the last column), the *P*/*N* ratio of *positive vs*. *negative* links (interactions) in the networks is an exception. The *P*/*N* ratio in the pre-chemotherapy sample (impacted by Hodgkin’s lymphoma) is approximately 10 times higher than those in the other two networks. This indicates that Hodgkin’s lymphoma may have a suppressing effect on the number of negative interactions in milk microbial networks. Some of the negative interactions involve opportunistic pathogens, and the breakup of the negative links may indicate the decline of inhibitive effects on the opportunistic pathogens in the pre-chemotherapy milk microbiome. The recovery of negative interactions in the post-chemotherapy demonstrates that chemotherapy seems to have a rehabilitating effect on the milk microbiome impacted by Hodgkin’s lymphoma.

Figs 1, 2, 3 exhibit the milk bacterial interaction networks of the pre-chemotherapy, post-chemotherapy and healthy microbiome samples, respectively. The above-described contrasting difference in *P/N* ratio is also obvious in Figs 1, 2, 3. There are only three negative interactions in the pre-chemotherapy network, compared with over 20 in the two other networks.

(*ii*) We further detected the basic motifs in the three networks with Cytoscape software, and the results are displayed in Table 2. It is shown that all of the basic motifs detected are most abundant in the healthy network, followed by pre-chemotherapy networks, and post-chemotherapy networks in a *decreasing* order. With existing dataset, we can only conjecture that Hodgkin’s lymphoma seemed to lower the number of motifs in the milk microbiome and the chemotherapy failed to stop the decline.

(*iii*) We further utilized the MCODE plug-in for Cytoscape^21^ to analyze the three networks by mining modules (clusters) that are more general than the basic motifs detected with standard Cytoscape package, because clusters detected by MCODE are not limited by the number of nodes or edges, as in the basic motifs displayed in Table 2. MCODE can detect *clusters*, which are highly interconnected regions in a network. In the three bacterial interaction networks we constructed for healthy, pre-chemotherapy and post-chemotherapy microbiomes, those clusters should represent OTU complexes that have high correlation coefficient values, which may signal some *functional groups* or *ecological guilds* in the underlying microbiome.

Table 3 lists the results of MCODE cluster-detection in the three bacterial interaction networks. For each network, we list the *number*, corresponding cluster *scores*, number of *nodes*, and number of *edges* for each cluster. The higher the score is, and the stronger the cluster is. From Table 3, we can see that the healthy network has a single strongest cluster with *16* nodes and *110* edges, and the other clusters are rather small with 3–4 nodes and 3–6 edges only. In contrast, the cluster strengths (score) in the diseased (pre- and post-chemotherapy) networks are relatively distributed evenly. For example, in the *pre-chemotherapy* network, the difference in the number of edges between the top two strongest clusters is only 11 (66 vs. 55), compared with the difference of 104 and 29 in the healthy and post-chemotherapy network, respectively. Table 3 also shows that the healthy microbiome network has a dominantly strong cluster, the diseased networks (pre- and post-chemotherapy) instead have 2–3 clusters with similarly moderate strength, and all three networks have variable numbers of small clusters with a minimum strength of approximately 1.

**Figure 4:**
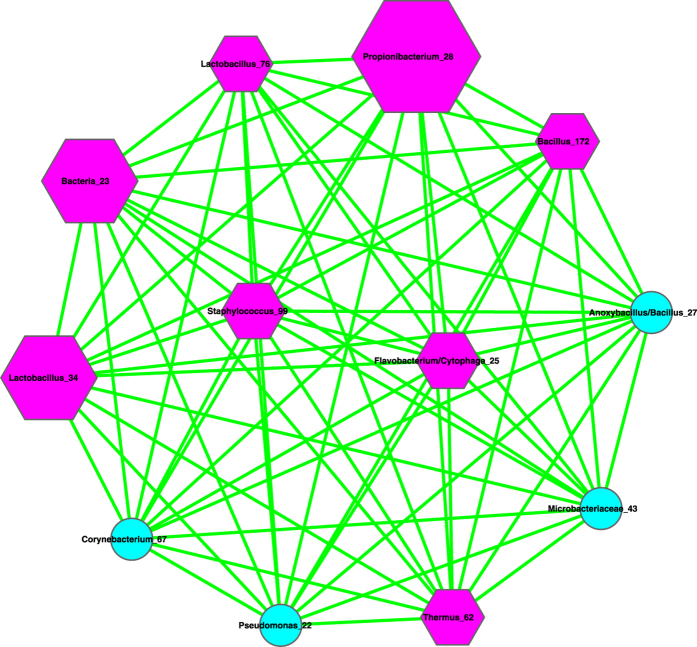
The strongest clusters in the milk bacterial interaction networks of the pre-chemotherapy samples, *i.e*., extracted from Fig. 1, and the symbols used are the same as in Fig. 1.

**Figure 5:**
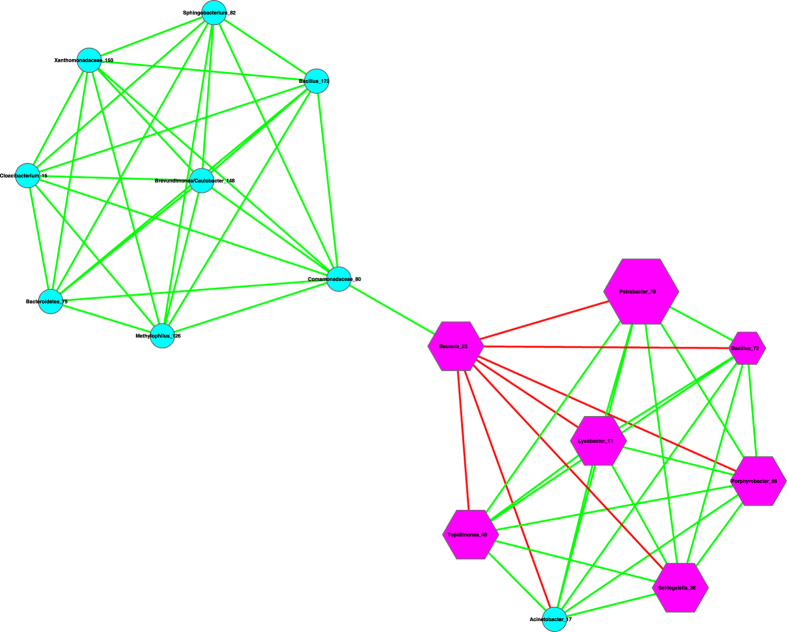
The strongest cluster in the milk bacterial interaction networks of the post-chemotherapy samples, *i.e*., extracted from Fig. 2, and the symbols used are the same as in Fig. 2.

**Figure 6:**
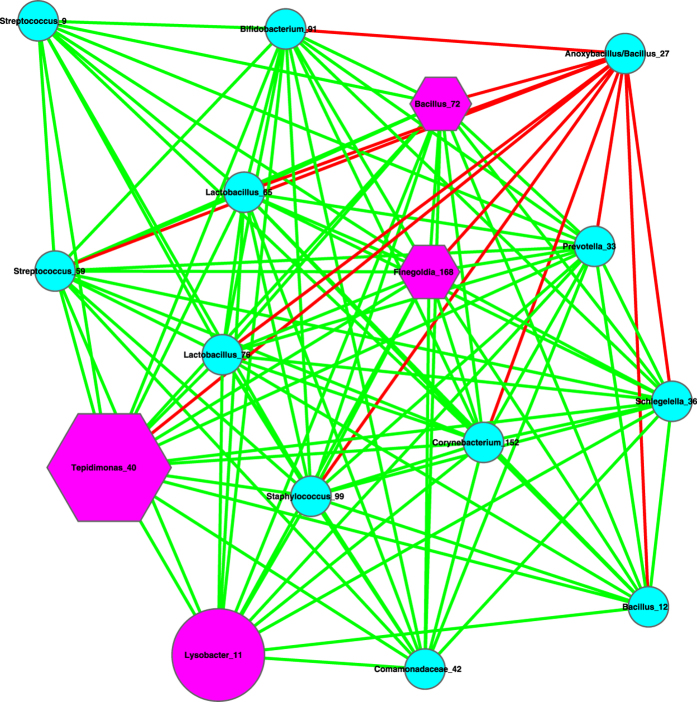
The strongest cluster in the milk bacterial interaction networks of the healthy samples *i.e*., extracted from Fig. 3, and the symbols used are the same as in Fig. 3.

Furthermore, the pattern of cluster distribution in the post-chemotherapy network is closer to the pattern in the healthy network than to the pattern in the pre-chemotherapy network. For example, in the healthy network, the ratio of the edge numbers between the top two strongest clusters is approximately *18*. In contrast, the ratios in the pre-chemotherapy and post-chemotherapy network are approximately 1 and 2, respectively. This again supports our previous conjecture, *i.e*., Hodgkin’s lymphoma may have a far-reaching influence on the milk microbiome by breaking up microbiome network into smaller and weaker clusters. The chemotherapy seems to be helpful for the milk microbiome to recover slightly by restoring the negative interactions, and therefore may possess a relieving effect on the milk microbiome impacted by the disease. There are not any negative links in the strongest cluster in the diseased pre-chemotherapy network (Table 4, Fig. 4), but negative links are abundant in the strongest clusters of the healthy and post-chemotherapy networks (Table 4, Figs 5 and 6).

### The comparisons of the community diversities among pre-chemotherapy, post-chemotherapy and healthy microbiomes

We computed the alpha diversities of the milk microbiomes from the three different groups of healthy, pre-chemotherapy and post-chemotherapy samples (Table 5). We further performed Student’s *t*-test to determine the difference among the three groups, and the results are listed in Table 6.

Table 6 shows that there are significant differences between the healthy and pre-chemotherapy samples or between the healthy and post-chemotherapy samples in terms of the alpha diversities at different orders. Therefore, Hodgkin’s lymphoma disease seems to have significant influence on the community diversity, but chemotherapy seems to have little influence on the community diversity. However, previous network analysis suggests that chemotherapy does influence milk microbiome network, with a possibly relieving effect for the milk microbiome, impacted by Hodgkin’s lymphoma, to recover.

On the surface, the lack of significant difference in the milk microbial community diversity (measured with the Hill numbers) between pre-chemotherapy and post-chemotherapy microbiome appears to contradict the difference in network properties between the pre-chemotherapy and post-chemotherapy microbiome, as suggested by the previous network analysis. A careful examination would readily reconcile this apparent contradiction. This is because community diversity is determined only by the species abundance distribution (SAD) in a community, and it does not reflect the interaction (measured in correlation) between species. Therefore, the lack of significant difference in diversity does not preclude the existence of other effects that chemotherapy may have on the microbiome. For example, the effect of chemotherapy on the interaction (correlation) among bacterial species, which is not reflected in the Hill numbers, may be reflected in the properties of milk bacterial interaction networks.

### The diversity-metabolites network (DMN) and metabolites-OTU network (MON)

**Figure 7:**
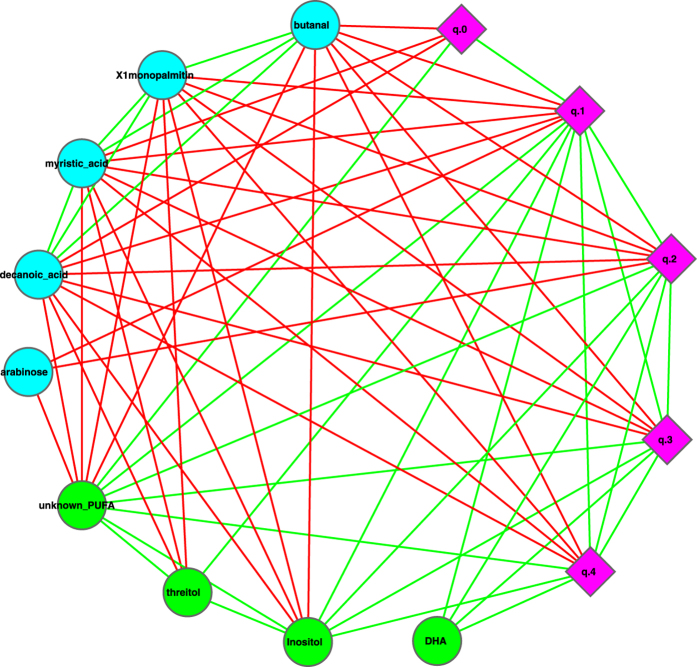
The metabolites-diversity network (MDN): symbols used: edges in green—positive correlation, edges in red—negative correlation, diamond—diversity at order *q* = 0–4, cycle in green—beneficial metabolite, cycle in cyan—unknown or potentially harmful metabolite.

**Figure 8:**
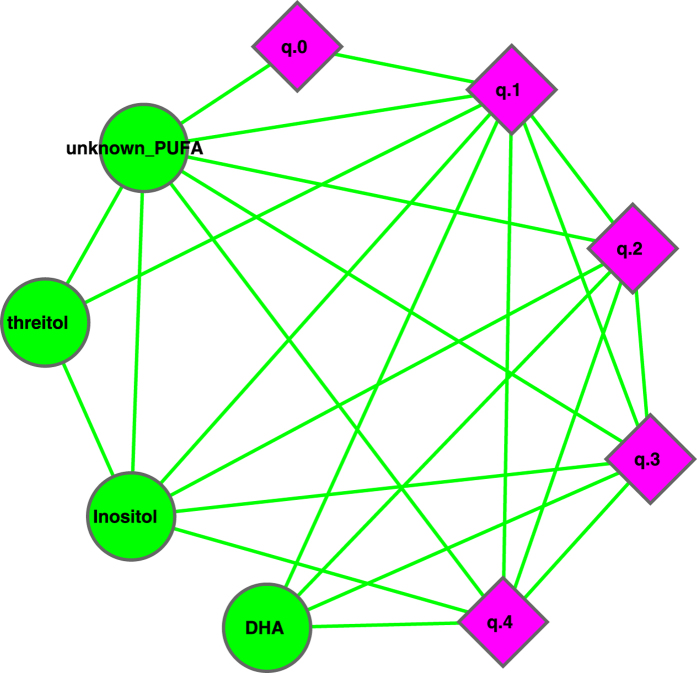
Sub-graph of MDN displaying the metabolites that are positively correlated with the diversity only, *i.e*., extracted from Fig. 7, and the symbols used are the same as in Fig. 7.

**Figure 9:**
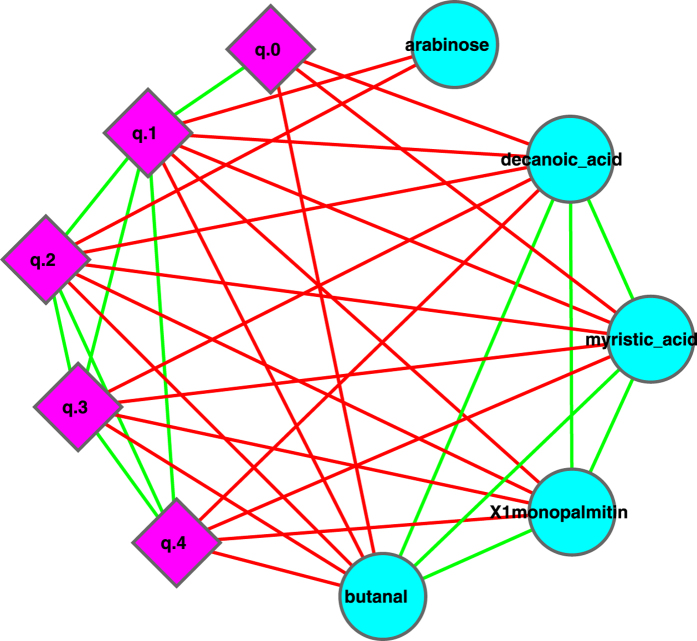
Sub-graph of MDN displaying the metabolites that are negatively correlated with the diversity only, *i.e*., extracted from Fig. 7, and the symbols used are the same as in Fig. 7.

We constructed a correlation network with the datasets of metabolites (abundances) and milk microbial community diversities at different diversity orders (*q*), and the resulting network graph is displayed in Figs 7, 8, 9. In Fig. 7, the network nodes can be classified into three types: the milk microbial community diversities measured in the Hill numbers from *0* to *4*^th^ order (5 nodes marked in pink diamond), 4 metabolites (*DHA*, *Inositol*, *Threitol*, and a *PUFA*) that are positively correlated with the Hill numbers, and 5 metabolites (*Butanal*, *Xlmonopalmitin*, *Decanoic Acid*, *Arabinose*, *Myristic acid*) that are negatively associated with the Hill numbers. Figures 8 and 9 are sub-graphs extracted from Fig. 7, which exclusively show the positively or negatively associated metabolites, respectively. The former includes the metabolites that are positively associated with the diversity only, and the latter includes the metabolites that are negatively associated with the diversity only. Figure 8 shows an interesting phenomenon that the diversity at order zero (*q*_0_) (*i.e*., species richness) is correlated with one metabolite (unknown PUFA) only. In contrast, the high-order diversity Hill numbers (*q* = 1–4) are closely associated with multiple metabolites and may be better indicators to the effects of the positively associated metabolites.

It seems worthy of particular notice that two metabolites positively associated with the diversity should be beneficial to baby’s health. For example, DHA is an unsaturated fat that plays a critical role in the development of baby’s eyesight and brain^26^,^27^. Inositol is a growth factor that lowers the cholesterol level. Furthermore, the effects of metabolites that are negatively associated with the diversity are unknown (Arabinose) or with lower toxicity (Myristic Acid)^28^. We hence postulate that higher microbial diversity in the milk microbiome is likely a ‘signature’ of healthy milk microbiome of healthy mothers, and therefore beneficial to infants. We further postulate that diseases, such as Hodgkin’s lymphoma that seems to lower milk microbial diversity, may have an opposite effect on mothers and their infants.

**Figure 10:**
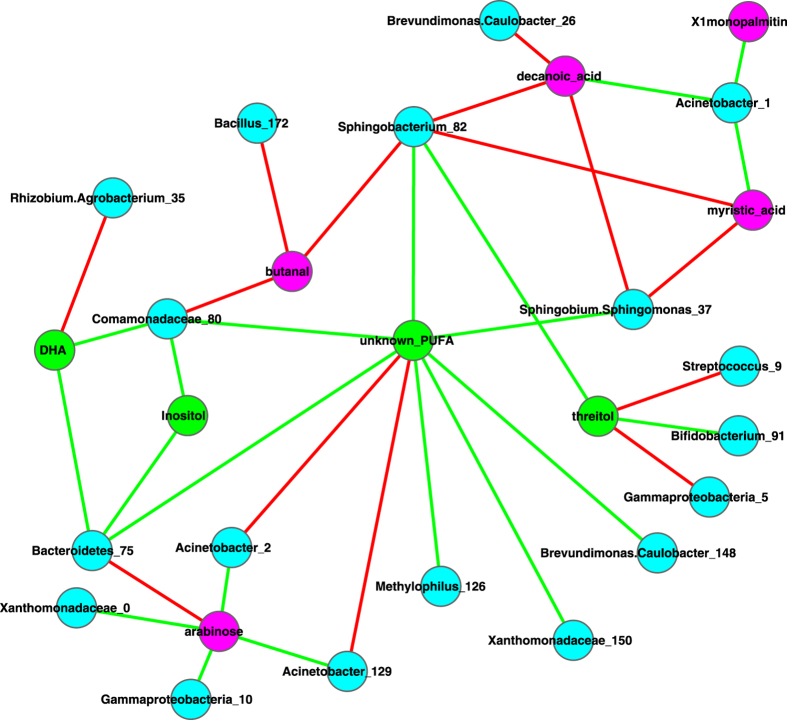
The metabolites-OTU (MON) network: symbols used: edges in green—positive correlation, edges in red—negative correlation, cycle in green—beneficial metabolite, cycle in pink—unknown or potentially harmful metabolite, and cycle in cyan—OTU.

Figure 10 further displays the metabolites-OTU network (MON), which is constructed based on the pair-wise correlation between OTU (nodes in cyan color) abundances and metabolite abundances (nodes in *pink* or *green*). The metabolites in Fig. 10 can be distinguished as two groups. One group consists of 4 nodes (green color) that are associated positively with diversity in Figs 7, 8, 9, and is associated with some beneficial bacteria in Fig. 10. In contrast, the other group consists of 5 nodes (pink color) that are associated negatively with diversity in Figs 7, 8, 9, and is associated with some potentially harmful bacteria in Fig. 10.

For example, three OTUs, *Acinetobacter_1, Acinetobacter_2 and Acinetobacter_129* of genus *Acinetobacter* are positively associated with potentially harmful metabolites, but negatively associated with beneficial metabolites. *Acinetobacter* is a genus including some opportunistic pathogens in the *proteobacteria* group^29^. *Acinetobacter baumannii,* one species of this genus, is often found as the cause of pneumonia in hospitalized patients, in particular those dependent on ventilators in the Intensive Care Units^30^. We conjecture that either the potentially harmful metabolites (Xlmonopalmitin, Decanoic acid, Myristic acid, and Arabinose) promote the growth of the three *Acinetobacter* OTUs, or they may be the metabolic products of the three OTUs. In addition, *Acinetobacter_2* and *Acinetobacter_129* exhibited negative correlation relationships with beneficial metabolite (a PUFA) (Fig. 10), and may be inhibited by the PUFA in healthy milk microbiome given their negative correlation.

For another example, many *Streptococcus* bacteria are opportunistic pathogens, and especially some group *B streptococci* cause life-threatening diseases in newborns, pregnant women, the elderly, and adults with compromised immune systems^31^. In Fig. 10, *Streptococcus*_9 is negatively associated with beneficial metabolite *Threitol*. We conjecture that it may by inhibited by *Threitol* in healthy milk microbiome.

Some other interesting relationships between milk bacteria and metabolites are also displayed in Fig. 10. *Comamonadaceae* exhibited positive correlations with beneficial metabolites (DHA & Inositol), and negative correlation with potentially harmful Butanal. *Comamonadaceae* are a family of the *Proteobacteria,* and they are Gram-negative and aerobic. *Bacillus* is negatively associated with *Butanal* (a potentially harmful meta-factor) and it constitutes 10% in the microbiome of breast tissue^10^. *Bacillus* is mostly beneficial bacteria and may play a role in suppressing the accumulation of Butanal.

### Summary

We reiterate the following findings summarized from the previous sections.

(*i*) Bacterial species interaction network (SIN) analysis shows that the ratio of positive *vs*. negative interactions (P/N ratio) in the *pre-chemotherapy* microbiome of Hodgkin’s lymphoma patient is approximately 10 times more than the P/N ratio in the *post-chemotherapy* microbiome of Hodgkin’s lymphoma patient, and is approximately 15 times more than the P/N ratio in the microbiome of *healthy* subjects. From this finding, we postulate that Hodgkin’s lymphoma may have a suppressing effect on the negative interactions in the milk microbiome, and the chemotherapy may have a relieving effect on milk microbiome by restoring the negative interactions. Given that many of the negative interactions involve opportunistic pathogens, the breakup of the negative links suggests the decline of inhibitive effects on the opportunistic pathogens in the diseased microbiome. The recovery of negative interactions in the post-chemotherapy microbiome suggests that chemotherapy may have a rehabilitating effect on the milk microbiome. Further network analysis with MCODE module detection technique also supports our postulation. That is, Hodgkin’s lymphoma has a far-reaching effect on the milk microbiome by breaking up the network into smaller and weaker clusters. The chemotherapy seems to be helpful for the milk microbiome to recover slightly.

(*ii*) Diversity analysis with the Hill numbers demonstrates that Hodgkin’s lymphoma has a significant influence on the milk microbial community diversities, but chemotherapy does not. Since diversity does not reflect the *interactions* between species, therefore the lack of influence from chemotherapy on community diversity does not imply that it does not have any effect on other aspects of microbiome, especially the interactions among microbial species, which is reflected in the findings from the previous network analysis.

(*iii*) Diversity-metabolites network (DMN) analysis suggests that the diversity is positively associated with some potentially beneficial metabolites such as DHA and Inositol, and negatively associated with some potentially harmful or unknown metabolites such as Butanal. From this finding, we postulate that high milk microbial diversity should be a *signature* of the healthy milk microbiome of healthy mothers, and hence beneficial to mothers and their infants.

(*iv*) Metabolites-OTU network (MON) analysis suggests that some OTUs may play critical role in the milk microbial community thanks to their close correlations with the metabolites. For example, some opportunistic pathogens such as three species of *Acinetobacter* genus and *Streptococcus*_9 are positively associated with some potentially harmful metabolites, and they are negatively associated with some potentially beneficial metabolites. We conjecture that beneficial metabolites may have inhibiting effects on those opportunistic pathogens. We also postulate that those potentially harmful metabolites may promote the growth of opportunities pathogens or they might be the products of the opportunistic pathogenic OTUs. For another example, mostly beneficial bacteria genus *Bacillus* is negatively associated with Butanal (a potentially harmful factor) and it constitutes 10% in the microbiome of breast tissue^10^, and we conjecture that *Bacillus* may inhibit the accumulation of potentially harmful Butanal.

Here we revisit the four questions raised in previous introduction section, and we also discuss the potential biomedical implications of those questions.

(*i*) *Do Hodgkin’s lymphoma and/or the chemotherapy for treating the disease add any significant ‘signature’ to the impacted milk microbiome compared with the healthy milk microbiome?* Our analyses revealed multiple contrasting differences between healthy and diseased microbiome samples, and one of the most conspicuous signatures should be the P/N ratio (positive vs. negative interactions). Since the P/N ratio may reflect the number of opportunistic pathogenic OTUs in milk sample, our finding of this ‘signature’ may help to assess the effect of Hodgkin’s lymphoma on the milk microbiome as well as its healthy implications to infants. A general treatment on the utilization of P/N ratio as an in silicon biomarker for differentiating between healthy and diseased microbiome is presented elsewhere by Ma et al. (2016, submitted).

(*ii*) *Does the chemotherapy have significant influence on the milk microbial diversity? If no, does it have any other important impacts that were missed by the diversity analysis?* Our diversity analysis failed to show the impact of chemotherapy on milk microbial diversity, but chemotherapy may affect other aspects of the microbiome such as species interactions. Indeed, chemotherapy appears to have a rehabilitating effect on the milk microbiome. The finding demonstrates an important methodological limitation, network or diversity analysis alone is not sufficient to fully reveal the influence of chemotherapy on the milk microbiome and an integrated approach of both analyses is necessary.

(*iii*) *How are the metabolites in the breast milk related to the milk microbial diversity?* Our diversity-metabolites network analysis suggests that the milk microbial diversity is positively correlated with some beneficial metabolites such as DHA and Inositol, and is negatively correlated with some potentially harmful metabolites such as Butanal. We postulate that high milk microbial diversity should be a *signature* of healthy milk microbiome of healthy mothers, and hence beneficial to mothers and their infants. To the best of our knowledge, this finding should be the first piece of quantitative evidence to support the health benefit of high bacterial diversity in the human milk.

(*iv*) *Are there any specific bacteria that are more closely associated with the important metabolites than their peers?* We identified that three OTUs of Acinetobacter genus, one OTU of Streptococcus and one OTU of Gemella may be opportunistic pathogens in milk microbiome, and all of them except for Gemella_47 appear to be negatively associated with beneficial metabolites and/or positively associated with potentially harmful metabolites. We further conjectured that beneficial metabolites might impose inhibiting effects on the opportunistic pathogens, or alternatively, potentially harmful metabolites might be the metabolic products of the opportunistic pathogenic OTUs. We also identified some beneficial bacteria (Bacillus and Comamonadaceae), which are negatively associated with potentially harmful metabolite Butanal; Comamonadaceae are also positively associated with beneficial metabolites (DHA & Inositol). These special OTUs obviously deserve further investigation.

Finally, we emphasize that the findings reported in this article are of preliminary nature, and many of our statements were formulated as postulations, due to the limitation of available datasets. Nevertheless, given the presently still very limited understanding of the milk microbiome, not to mention answering the four specific questions we raised previously, the results we obtained are of significant biomedical implications. To the minimum, the postulations we proposed as well as the OTUs we identified are worthy of further investigations obviously.

## Additional Information

**How to cite this article**: Ma, Z. *et al*. Integrated network-diversity analyses suggest suppressive effect of Hodgkin’s lymphoma and slightly relieving effect of chemotherapy on human milk microbiome. *Sci. Rep.* **6**, 28048; doi: 10.1038/srep28048 (2016).

## Figures and Tables

**Table 1:** The basic properties of the milk bacterial interaction networks.

Networks	Num. of Nodes	Num. of Edges	Average Degree	Avg. Local Cluster Coefficient	Diameter	Average Path Length	Num. of Commu-nities	Network Density	Network Modularity	P/N Ratio = Positive/Negative
Pre-Chem.	69	273	7.913	0.734	10	4.219	6	0.116	0.690	90 (270/3)
Post-Chem.	69	206	5.971	0.660	11	4.449	7	0.088	0.719	9.3 (180/20)
Healthy	60	209	6.967	0.636	9	3.792	9	0.118	0.399	6.2 (180/29)

**Table 2:**
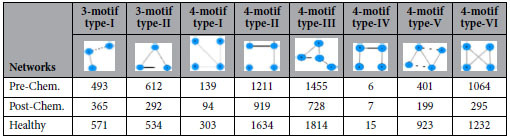
The number of basic motifs detected in the milk bacterial interaction networks.

**Table 3:** The most strongly connected modules (clusters) in the healthy, pre-chemotherapy and post-chemotherapy networks.

Healthy network				Pre-chemotherapy network				Post-chemotherapy network			
*Cluster No.*	*Score*	*Nodes*	*Edges*	*Cluster No.*	*Score*	*Nodes*	*Edges*	*Cluster No.*	*Score*	*Nodes*	*Edges*
1	6.875	16	110	1	5.5	12	66	1	3.562	16	57
2	1.5	4	6	2	5	11	55	2	3.5	8	28
3	1.25	4	5	3	4	9	36	3	2.5	6	15
4	1	3	3	4	2.5	6	15	4	2.444	9	22
5	1	3	3	5	2	5	10	5	1.5	4	6
				6	1	3	3	6	1.5	4	6
				7	1	3	3	7	1.25	4	5
				8	1	3	3				
				9	1	3	3				

**Table 4:** The negative links distributed in the strongest clusters in the three networks.

Network	Species 1	Species 2
Healthy Network	*Anoxybacillus/Bacillus_27*	*Bacillus_12*
	*Anoxybacillus/Bacillus_27*	*Prevotella_33*
	*Anoxybacillus/Bacillus_27*	*Schlegelella_36*
	*Anoxybacillus/Bacillus_27*	*Tepidimonas_40*
	*Anoxybacillus/Bacillus_27*	*Streptococcus_59*
	*Anoxybacillus/Bacillus_27*	*Lactobacillus_65*
	*Anoxybacillus/Bacillus_27*	*Bacillus_72*
	*Anoxybacillus/Bacillus_27*	*Lactobacillus_76*
	*Anoxybacillus/Bacillus_27*	*Bifidobacterium_91*
	*Anoxybacillus/Bacillus_27*	*Staphylococcus_99*
	*Anoxybacillus/Bacillus_27*	*Corynebacterium_152*
	*Anoxybacillus/Bacillus_27*	*Finegoldia_168*
Pre-Chemotherapy Network	None	None
Post-Chemotherapy Network	*Bacteria_23*	*Lysobacter_11*
	*Bacteria_23*	*Schlegelella_36*
	*Bacteria_23*	*Tepidimonas_40*
	*Bacteria_23*	*Porphyrobacter_66*
	*Bacteria_23*	*Bacillus_72*
	*Bacteria_23*	*Petrobacter_79*
	*Bacteria_23*	*Acinetobacter_17*

**Table 5:** The alpha diversity (measured in Hill numbers) of the pre-chemotherapy, post-chemotherapy, and healthy milk microbiome samples.

Network	Sample ID	*q* = 0	*q* = 1	*q* = 2	*q* = 3	*q* = 4
Pre-chemotherapy samples	0A	57	19.264	10.856	8.280	7.172
	2A	59	8.227	3.185	2.487	2.257
	4A	59	7.357	4.389	3.644	3.307
	6A	57	9.131	5.772	4.910	4.534
	10A	53	11.532	7.756	6.406	5.715
	12A	51	3.063	2.078	1.867	1.770
	14A	54	10.756	6.167	5.000	4.487
	16A	51	1.514	1.178	1.133	1.117
Mean		55	8.856	5.173	4.216	3.795
Post-chemotherapy samples	0B	61	28.335	20.045	16.248	14.188
	2B	61	11.679	4.406	3.214	2.833
	4B	56	11.432	6.827	5.530	4.938
	6B	48	5.863	3.872	3.389	3.165
	10B	54	11.055	7.525	6.671	6.308
	12B	46	2.851	2.187	2.056	1.986
	14B	57	6.996	4.408	3.651	3.303
	16B	55	7.533	3.871	3.114	2.811
Mean		55	10.718	6.643	5.484	4.942
Healthy samples	H1	46	22.130	14.242	11.362	10.030
	H2A	42	15.721	10.483	8.897	8.149
	H2B	44	18.051	10.976	8.642	7.584
	H3	50	20.789	14.849	12.792	11.775
	H5	42	20.522	14.271	11.929	10.777
	H6	52	22.699	14.082	10.243	8.422
	H7	44	19.175	11.909	9.591	8.564
	H8	46	18.674	11.846	9.465	8.314
	H10	48	15.379	8.988	7.048	6.189
Mean		46	19.238	12.405	9.997	8.867

**Table 6:** The results of Student’s *t*-test of the Hill numbers among healthy, pre-chemotherapy and post-chemotherapy microbiome samples.

Diversity Order (*q*)	Healthy *vs*. Post-chemotherapy			Healthy *vs*. Pre-chemotherapy			Pre-chemotherapy *vs.* Post-CT		
	Healthy	Chemo-	*p-value*	Healthy	Chemo-	*p-value*	Post-C	Pre-C	*p-value*
0	46.000	53.857	0.000	46.000	54.857	0.000	53.857	54.857	0.681
1	19.238	8.201	0.000	19.238	7.369	0.000	8.201	7.369	0.670
2	12.405	4.728	0.000	12.405	4.361	0.000	4.728	4.361	0.751
3	9.996	3.946	0.000	9.996	3.635	0.000	3.946	3.635	0.746
4	8.867	3.621	0.000	8.867	3.312	0.000	3.621	3.312	0.723
